# Emotional self-regulation and personality in the light of Thomas Aquinas’s philosophical anthropology

**DOI:** 10.3389/fpsyg.2024.1419202

**Published:** 2024-05-31

**Authors:** Juan Pablo Rojas-Saffie, Nicolás García-Matte

**Affiliations:** ^1^Department of Psychology, Faculty of Education, Psychology and Family, Universidad Finis Terrae, Santiago, Chile; ^2^Center for Research in Education, Psychology and Family (CIPEF), Faculty of Education, Psychology and Family, Universidad Finis Terrae, Santiago, Chile; ^3^Millenium Institute for Depression and Personality Research (MIDAP), Doctoral Program in Psychotherapy, Faculty of Social Sciences and Faculty of Medicine, Pontificia Universidad Católica de Chile and Universidad de Chile, Santiago, Chile

**Keywords:** emotions, affectivity, self-regulation, five-factor model, virtues, Thomistic anthropology, Integral psychology of the person

## Abstract

This article aims to thoroughly understand the concept of emotional self-regulation (ESR) and its relationship with personality. Through an interdisciplinary dialogue between psychology and philosophy—specifically, the anthropology of Thomas Aquinas—three realities are proposed that could be considered as ESR. The conceptual relationship between ESR—understood as *operation*, *faculty* and *habit*—and personality is examined, specifically using the Five-Factor Model and the virtues model. Key findings include the need for consensus on a precise definition of ESR, the central role of reason as a faculty capable of ruling over emotions, the relevance of the distinction between ESR and self-control, and the understanding of ESR as a set of habits that include aspects of *prudence*, *temperance* and *fortitude*. Interdisciplinary dialogue seems to be a valuable intellectual approach to the advancement of the field of psychology.

*At all events we may firstly observe in living creatures both a despotical and a constitutional rule; for the soul rules the body with a despotic rule, whereas the intellect rules the appetites with a constitutional and royal rule. And it is clear that the rule of the soul over the body, and of the mind and the rational element over the passionate, is natural and expedient; whereas the equality of the two or the rule of the inferior is always hurtful (*Aristotle, 1916, *Politics, Book I, Chapter 5)*.

## 1 Introduction

Emotional self-regulation (ESR) is a relatively recent notion within academic psychology. Although there is some prior history, the field of emotional regulation emerged strongly only in the mid-1990s (Gross, 2015a). However, the idea that we can influence our emotions has been visible in Western thought for almost 2,500 years. For instance, Aristotle (1916, Book I, Chapter 5) proposed that human beings can exercise political mastery over their emotions, which is indispensable for achieving virtue and happiness.

An important aspect of this concept is inter-individual differences. Studies, as well as experience, show that people differ in their skill to manage their emotions. This is seen between people of different ages (e.g., Orgeta, 2009) and adults of the same cohort (e.g., Gross and John, 2003; Olderbak et al., 2023). While, at one end, some barely regulate their emotions and impulses, at the other, there are individuals who lack spontaneity due to their constant efforts to keep emotions under control. However, it can also be observed that many manage to control their affective world in an appropriate way. These differences constitute different stable patterns of behavior and can therefore be considered within the realm of personality.

Although the link between ESR and personality has already been addressed (e.g., Baumeister et al., 2006; Hoyle, 2006, 2010; Morf, 2006; McCrae and Löckenhoff, 2010), its nature remains unclear, as can be seen in the difficulty that arises when trying to answer what is ESR and how is its relationship with personality. Regarding the first question, the heterogeneity of definitions of ESR found in the scientific literature is striking, as will be shown later. It’s unclear whether ESR is a common capacity or a skill that can be developed, as a personality domain (Roberts and Yoon, 2022). If we conceive ESR as a common capacity, then it would be independent of personality since the latter designates aspects in which people are unique (Allport, 1937). However, given that correlations have been shown between personality traits and ESR (Hoyle, 2006, 2010; McCrae and Löckenhoff, 2010), perhaps it is a skill. The problem is that if it is linked to certain traits, and the traits are biologically based and unintentionally developed according to the Five Factor Model (FFM) (Fowers et al., 2023), then we would have to conclude that ESR is exclusive to some personality types. Nevertheless, this is against the general belief that all people are capable of developing ESR toward a mature and healthy personality (Allport, 1937; Arnold, 1960).

The relationship between ESR and personality also poses difficulties. It is unclear whether ESR is a personality trait or a meta-trait situated “above” (Strauman and Wilson, 2010; Fowers et al., 2023). If it is a trait, it would be a unique one, because there is no other trait directly committed to the modulation and expression of the others. Moreover, if we consider it a meta-trait, we would have to consider it independently from personality, which seems contradictory to the evidence that some personality traits are more closely related to ESR (Hoyle, 2006, 2010; McCrae and Löckenhoff, 2010). Furthermore, it is not clear what psychological structure would be able to contain this meta-trait. Finally, people who successfully regulate their emotions distinguish themselves affectively and behaviorally from others, which is proper to the notion of personality. Therefore, it seems counterintuitive not to include ESR in personality.

Engaging in an interdisciplinary dialogue between psychology and philosophical anthropology, specifically Thomistic anthropology, seems appropriate to adequately address these questions. The psychological literature has come a long way in understanding self-regulation but has yet to reach a cross-cutting terminological agreement, let alone a consensus on its exact meaning. Philosophical anthropology, on the other hand, has a particular interest in understanding the nature of psychic phenomena and their appropriate formulation; indeed, it attempts to answer the ultimate questions about the human being (Asociación de Psicología Integral de la Persona, 2022; Redshaw and Ganea, 2022). In particular, the work of Thomas Aquinas seems quite suitable for addressing the topic of ESR since it offers a comprehensive theoretical framework about the human being, its structure, the relationship between its faculties, and the integration of rational and sensible aspects. In fact, the coherence and usefulness of his ideas has recently been verified in an article that analyzed the *Extended Process Model of the Emotion Regulation* proposed by Gross (1999) in the light of Aquinas’s work (Marple et al., 2024). Furthermore, the Thomistic schema seems particularly appropriate because it provided the basis for *appraisal* theory (Arnold, 1960), which has been fundamental both for constructing different models of ESR (e.g., Lazarus, 1973; Gross, 2024) and also for exploring individual differences in emotion (e.g., Kuppens and Tong, 2010; Stemmler and Wacker, 2010).

In order to achieve the fruitfulness of this interdisciplinary dialogue, we will start by reviewing the scientific literature devoted to ESR. We will then introduce some Thomistic postulates, reviewing how they allow us to understand and classify the postulates in the literature. Finally, we will address the link between self-regulation and personality, considering the five-factor model (FFM) and the virtues model of the Aristotelian-Thomistic tradition.

## 2 Emotional self-regulation in psychological literature

Despite having its roots in developmental psychology, the concept of self-regulation emerges strongly at the end of “the era of radical behaviorism, [when] ‘self-regulation’ and other designations for the concept of will were banned from experimental psychology as ‘unscientific’” (Kuhl, 2018, p. 542). This is how self-regulation begins to be considered a key mechanism in the interactionist perspective of *social cognitive theory,* concerning human agency (Bandura, 1991) and personality dynamics, where it describes how the motivation to achieve certain goals requires the self-regulatory force of will (Mischel and Ayduk, 2004). Thus, in these theories, self-regulation is understood as a mechanism that allows human beings to change their behavior, thoughts and emotions based on hierarchically organized norms, goals and standards of interaction (Carver and Scheier, 2001). Along these lines, some models have described the concept of self-regulation as an adaptive personality trait (Baumeister et al., 2006) or as a set of natural and adaptive actions of healthy individuals (Hoyle, 2010). However, emotional self-regulation only corresponds to a part of the general system described above. At the same time, it is nourished by the psychoanalytic tradition of psychic conflict between drives and external factors and by studies on stress and coping strategies (Gross, 1999).

In recent years, ESR has expanded strongly in the field of psychology (McRae and Gross, 2020) because at its core lies an idea that has been central to the Western view of mental health (Shanker, 2012) and to the ongoing maintenance of psychological well-being (Doré et al., 2016): we do not experience our emotions as passive observers, but actively influence them (Gross, 1999). This active conception of emotion has its roots in the moral philosophy of ancient Greece, from where it evolved throughout history and was incorporated into the medieval scholastic synthesis expounded by Thomas Aquinas in the 13th century. It was also one of the first contributions of Magda Arnold (1960, 1973) to the field of cognitive emotion theory, integrating her experimental work with the conceptual framework of Thomistic psychology (Cornelius, 2006), as some authors have recently done (e.g., Lombardo, 2011; Gross et al., 2020).

Greatly influenced by Aquinas, Arnold (1960) proposed a cognitive theory of emotion whose major and most revolutionary contribution is to consider that every emotional response is preceded by an intuitive, momentary and personal judgment that evaluates the situation, called *appraisal* (Arnold and Gasson, 1954; Arnold, 1973). This concept stems from Thomistic anthropology, which proposes an internal sense called *cogitative*, which alludes to the faculty that evaluates what is perceived as convenient or harmful in the light of the individual’s vital interests and from whose evaluation the affective movement arises (Aquinas, 1920, I, c. 78, a. 4). In this sense, an “emotion is not something that happens to us but something we do” (Arnold, n.d., p. 7, cited in Cornelius, 2006, p. 978).

While Arnold considered that emotions tend to help a person achieve his goals, they can sometimes get in the way of the *self-ideal*, so she proposed that *emotional control* could help a person not only to reduce or restrict their emotions, but to manage them in order to achieve an effective organization of the personality (Arnold and Gasson, 1954; Arnold, 1960). By strongly linking motivation to personality development, she considered that *emotional control* is only possible and necessary if there are goals that merit going against emotion, in that “emotional control means both a turning toward what is truly lovable from a human point of view and a turning away from things that exert too strong a pull” (Arnold, 1960, p. 278) through the use of reason. In this way, Arnold linked *emotional control* to personality development, integrating the contributions of Allport, Goldstein, and Maslow in her extended *appraisal theory* (Cornelius, 2006).

Richard Lazarus extended and deepened Arnold’s *appraisal* concept (Moors et al., 2013), differentiating it from knowledge (Lazarus, 1999), by being more explicit in the motivational component of emotions (Reisenzein, 2006), linking the constructs of emotion and stress (Lazarus, 1991), and including the possibility that the person can *re-appraise* the situation, to maintain some control over the emotion —*emotion-focused coping*—, or the situation —*problem-focused coping*— (Lazarus, 1973). He thus argued for the interdependence of cognition, motivation and emotion in all person-environment relationships (Lazarus, 1999) and proposed that appraisal is influenced by circumstances in interaction with personality variables such as goals and beliefs (Smith and Lazarus, 1990). Lazarus managed to systematize Arnold’s contributions from important observations that highlight the importance of ESR (Koole and Aldao, 2016), as he expressed in his early writings on the subject: “I have deliberately used the expression, ‘self-regulation’ to convey the theme that it is the person, appraising the personal and social requirements of an emotional situation, who manages his emotional reactions willfully” (Lazarus, 1973, p. 176).

*Appraisal* theory represents only one of the perspectives within the continuum of theories of emotion proposed in the field of psychology (Gross and Feldman Barrett, 2011). However, its singularity lies in the fact that it allows us to understand both the processes of emotion generation and emotion regulation, whose research has been surprisingly separate (Smith and Kirby, 2011) until their integration into a unified perspective (Yih et al., 2019) through Gross’s (2015b) model of emotion regulation, possibly one of the most comprehensive at present (Hughes et al., 2020).

Indeed, Gross (1998, 2015a, 2024) has systematically contributed to the understanding of emotional regulation from the semantic distinction of other related concepts and from the description of the process itself. Gross (2024) coined the concept of affective regulation to include, in addition to emotional regulation, *mood regulation, coping or stress regulation, and self-control* of impulses. From the *appraisal* theory, Gross (2015b) understands emotional regulation as a process that seeks to increase (*up-regulate*) or decrease (*down-regulate*) the intensity, duration and/or quality of an emotion valued personally as good or bad in a particular situation. In this way, he proposes that emotional regulation is driven by goals that, although they may be explicit or implicit (for a review, see Gyurak et al., 2011), healthy or unhealthy (for a review, see John and Gross, 2004), are aimed at changing the emotion of another individual (*extrinsic emotion regulation or interpersonal regulation*, e.g., Niven et al., 2024) or the emotion of oneself (*intrinsic emotion regulation*), which is what he understands as *emotional self-regulation*.

Gathering the contributions of different emotion theorists (e.g., Arnold, 1960; Lazarus, 1991), Gross (2008) proposes the *Modal Model of Emotion*, from which he describes the mechanisms behind emotion as an iterative and cyclical sequence of four elements: situation, attention, appraisal and response. In addition, he proposes the *Extended Process Model of the Emotional Regulation* (see Gross, 2015b) to describe different stages, such as identification, strategy selection, implementation and monitoring. For Gross (2008), individual differences in emotional regulation seem to lie in the choice of regulatory strategies, which may consist of a selection or modification of the situation, an attentional deployment, a cognitive change — such as *re-appraisal*—, or a modulation of the response — as *expressive suppression*—.

In this regard, Koole (2009) proposes an organization of ESR strategies according to functions that may combine or conflict: Need-Oriented Strategies, focused on seeking pleasure and avoiding pain with a hedonic or adaptive goal (e.g., attentional avoidance and stress-induced eating); Goal-Oriented Strategies, focused toward the accomplishment of specific tasks or goals (e.g., reappraisal and suppression); and Person-Oriented Strategies, oriented toward the achievement of several whole-personality goals (e.g., meditation and expressive writing), seeking “integration, which is manifested in the coordinated functioning of personality systems traditionally considered antagonistic, such as positive vs. negative emotions, body vs. mind, passion vs. reason, and top-down vs. bottom-up processing” (Koole, 2009, p. 26). For this reason, two Person-Oriented Regulation Models will be reviewed, which are particularly interesting because they delve into the relationship between ESR and personality, as they propose a way to coordinate the overall functioning of the personality or *self*, promoting its flexibility, coherence and growth (Koole and Aldao, 2016).

In the first place, Personality Systems and Interactions (PSI) theory (Kuhl, 2000, 2018) “is an integrative framework that seeks to explain human personality functioning in terms of its underlying functional mechanisms” (Kuhl and Koole, 2004, p. 421), including the central role of will and the mediating role of emotion. Building on the Aristotelian theory of motivation (Kuhl, 2000) and considering will as a top-down regulation mechanism, the model distinguishes between two volitional forms as the highest level of personality organization (Kuhl et al., 2021). The first, self-control, is understood as an explicit, conscious and effortful system that only responds to one goal at a time, metaphorically identified with an “inner dictatorship” (Kuhl and Koole, 2004), similar to Goal-Oriented Strategies (Koole, 2009). The second, self-maintenance, also called self-regulation, is a system capable of responding to goals that simultaneously satisfy a variety of aspects, thus resembling an “inner democracy” (Kuhl, 2018) and the Person-Oriented Strategies (Koole, 2009).

For Frijda (2013), the most valuable aspect of PSI theory is that it functionally understands regulation as effortful, voluntary, intentional and freely chosen. However, Kuhl et al. (2021) explain that these characteristics only define self-control, as it is an explicit processing system. On the contrary, self-regulation would operate at an implicit experiential level of personality integration toward a coherent identity (Kuhl et al., 2021), similar to the identified and integrated regulations of Self-Determination Theory (STD) (Ryan and Deci, 2019), where the person acts voluntarily driven by the value of the activity, first identified and then coherently integrated with the rest of the values. Along these lines, a distinction has emerged between *effortful* and *effortless willpower*, corresponding to self-control and self-regulation, respectively (see Quirin et al., 2021).

In the second place, the Strength Model of Self-Regulation (SMSR) (Baumeister, 2016; Baumeister and Vohs, 2016) proposes that self-control or self-regulation — used interchangeably by Vohs and Baumeister (2004) as regulation implies regular control — works like a muscle that can be exercised, but also fatigues when the available energy runs out (Baumeister et al., 2016). To understand its link to social relations, Baumeister and Exline (1999) designate self-control as the Master Virtue, in that the cardinal virtues described by Aquinas —prudence, justice, temperance and fortitude — would be based on the positive exercise of self-control and its main ingredients (standards, monitoring and operations), which in turn promote prosocial behaviors. However, Fowers (2008) comments that as long as the focus is on the control of desires, this position would be proper of a moral continence, while in the exercise of virtue, emotions and motivations are expressed adequately from the beginning of the action.

In their relation to personality, Baumeister and Exline (1999) explain that self-control is an operation that allows orienting desires toward the culture’s standards and is, therefore, a capacity shared by people to consciously or automatically (in the case of virtues) exercise their willpower. “Translated into personality theory, this view implies that people have stable differences in their capacity for exerting self-control to achieve virtuous actions” (Baumeister and Exline, 1999, p. 1179), which in particular is observed in the self-consciousness, the monitoring, the pursuit of virtuous behavior, and the “moral muscle” strength, vulnerable to be depleted.

This ego-depletion effect has been strongly criticized by several studies (e.g., Dang, 2018), but the lack of conclusive evidence (Friese et al., 2019; Englert and Bertrams, 2021), continues the debate and the search for more robust explanations, such as those based on the use of glucose to moderate energy expenditure (Baumeister et al., 2016). Valued by Baumeister and Vohs (2016) — and criticized by others (e.g., Inzlicht and Marcora, 2016; Bertrams, 2020) — the creative proposal of a “central governor” (see Evans et al., 2016) resembles a central bank or monetary authority in a liberal regime, concerned with an economic outlook for energy resources, in that they uphold the regulation of all operations.

Throughout the literature review of the concept of ESR, two major difficulties have emerged in its study in relation to personality. First, a large number of concepts have been proposed to indicate aspects related to emotion regulation, the most important being self-control and self-regulation, but even these have not been properly defined or distinguished in a transversal way in the field of psychology. As Quirin et al. (2021) puts it, “without a clear theory for drawing distinctions, neglecting to distinguish between effortful and effortless willpower (i.e., self-control vs. self-regulation) may more likely” (p. 3), and thus the study of their relationship to personality becomes more complex. For instance, while some authors make clear distinctions between the concepts of self-regulation and self-control (e.g., Kuhl et al., 2021; Gross, 2024), others use them interchangeably (e.g., Lazarus, 1991; Baumeister and Exline, 1999) or allude to them with other concepts, such as emotional control (e.g., Arnold, 1960).

Second, much of the research on personality differences has focused more on describing ESR processes and strategies rather than addressing the motivations that explain interindividual differences, i.e., the focus has been on describing how people regulate their emotions, and less on why they do it in a certain way and not in another (Tamir, 2016; Hughes et al., 2020). Based on this question, distinguishing between goals — desired emotional states (e.g., less sadness) — and motives — desired outcomes (e.g., doing well on an exam) — in emotion regulation helps to understand more clearly the process behind selection and, in turn, attending to the taxonomy that distinguishes between the different motives for regulating emotions—hedonic, directed at the emotion itself, vs. instrumental, oriented toward its possible benefits—, allows for a deeper understanding of the mechanisms of emotion regulation (for a review, see Tamir, 2016). From this taxonomy, the Big Five has been exposed as the ideal model for predicting ESR goals typically pursued according to personality traits (Kobylińska and Kusev, 2019), including, for instance, the positive association between Neuroticism and impression management goals related to the image an individual wants to project to others (Eldesouky and English, 2019). It has also been evidenced that the stages of identification, selection and implementation of ESR (Gross, 2015b) are strongly related to the Big Five taxonomy (for a review, see Barańczuk, 2019; Hughes et al., 2020).

This focus goes back to the contributions of Aristotle and Thomas Aquinas to understand the relationship of emotional regulation to ethical virtues, the latter being also known as habitual dispositions of character, which are context-sensitive and goal-oriented. Indeed, emotional regulation has been defined as a skill that is part of the cultivation of virtue (Carron, 2022; Krettenauer and Stichter, 2023), as well as one of the four primary functions of the virtue model of *phronesis* or *practical wisdom*, specifically linked to empathy and perspective-taking (Darnell et al., 2022). Thus, the level of emotional regulation would be a sign of the excellence of the virtuous person’s character, whose emotional harmony allows him to approach the good life of *flourishing* (Fowers, 2008), an ultimate sense of well-being that can be related to a specific type of ESR: mentalized affectivity (Jurist et al., 2023). Thomistic theories of emotional generation and types of appetite have even been integrated with the *Extended* *Process Model of the Emotion Regulation* by Gross (2015b), in particular the strategy of *re-appraisal*, finding among its results the importance of people using their will correctly (Marple et al., 2024). Thus, in recent years, there has been an increased interest in bringing the philosophical thought of Aristotle and Aquinas into dialogue with psychology due to its potential to understand the human being in a unified way, overcome the fragmentation of the discipline (Spalding et al., 2019) and clearly distinguish the different concepts.

Aristotelian-Thomistic philosophical anthropology states that living beings possess a vital principle called soul (Aquinas, 1920, I, c. 75, a. 1). Plants possess a vegetative soul, which is the vital principle of plant operations, such as feeding, growing and reproducing. Animals possess a sensitive soul, which enables them to feel, appetite and move. Finally, humans possess a rational soul, which allows us to understand and desire. For these authors, the higher levels of life assume the perfections of the lower levels, which would explain why animals are also capable of the operations of plants without having to resort to a duplicity of souls. The same is true of human beings, in whose rational soul the vegetative, sensitive and rational dimensions can be distinguished, without losing their uniqueness (1920, I, c. 76, in c.).

Of particular interest for the understanding of ESR is the distinction between the sensitive and rational dimensions of the human soul. For Aquinas (1920, I, c. 78, a. 1; a. 2; a. 3; a. 4), there are some faculties that are common to humans and animals, such as the capacity to feel stimuli –*external senses*–, to integrate them, to store them, to value them –*internal senses*– and to emotionally react to them –*sensitive appetite*–. This affective response, called *passion*^1^, consists of an inclination toward what is perceived as convenient, and a consequent rejection of what is perceived as harmful. On the other hand, there are some exclusive faculties in human beings, such as the capacity to understand and to become aware –*reason*–, and the capacity to love the intangible good and to make free choices –*will*–, as can be seen in Table 1.

**Table 1:** Thomas Aquinas’s scheme of human faculties.

Psychic dimension	Cognitive faculties		Appetitive faculties	
Rational dimension	Reason	Faculty that allows the human being to understand, reason and rule over emotions.	Will	Faculty that allows the human being to tend toward the intangible good and make choices.
Sensitive dimension	Internal senses	Memory: faculty that stores images in terms of lived experiences.	Sensitive appetite	Concupiscible appetite: faculty that tends toward tangible good, insofar as delectable.
		Cogitative: faculty that evaluates images as convenient or harmful.		
		Imagination: faculty that forms the internal image of the external stimulus.		Irascible appetite: faculty that tends toward the tangible good, insofar as arduous.
		Common sense: faculty that integrates the information of the stimuli.		
	External senses	Faculties oriented to sense the external world, such as touch, taste, smell, hearing, and sight.		

**Figure 1:**
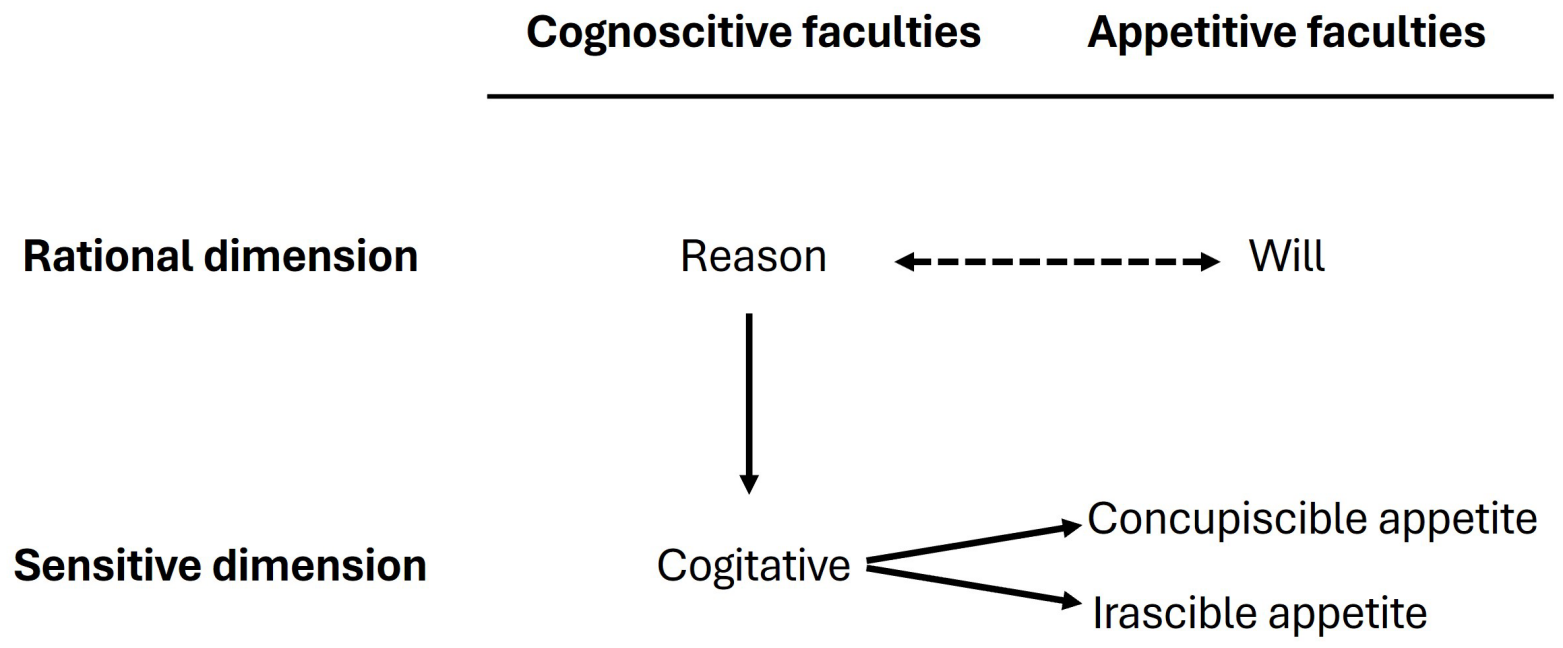
Reason commands over sensitive appetites through the *cogitative*. Reason can be moved by the will, and vice versa.

For Aristotle and Thomas Aquinas, human beings reach fullness to the extent that they live according to their reason and driven by their emotions, for which it is necessary to admit a kind of dominion over their passions (Botkin, 1921). Aquinas affirms that the sensitive appetite is naturally receptive to the command of reason (1920, I, c.81, a. 3; 1999, a. 8) through the inner sense called *cogitative*. Reason, by its universal type of cognition, moves this internal sense to carry out a particular cognition, thus triggering the movement of the sensitive appetite in the form of emotion (Aquinas, 1920, I-II, c. 17, a. 7), as can be seen in Figure 1.

## 3 Interdisciplinary dialogue

To facilitate the dialogue, we have structured the interdisciplinary analysis around two main questions: (1) What is ESR? and (2) How is its relationship with personality?

### 3.1 What is ESR?

**Figure 2:**
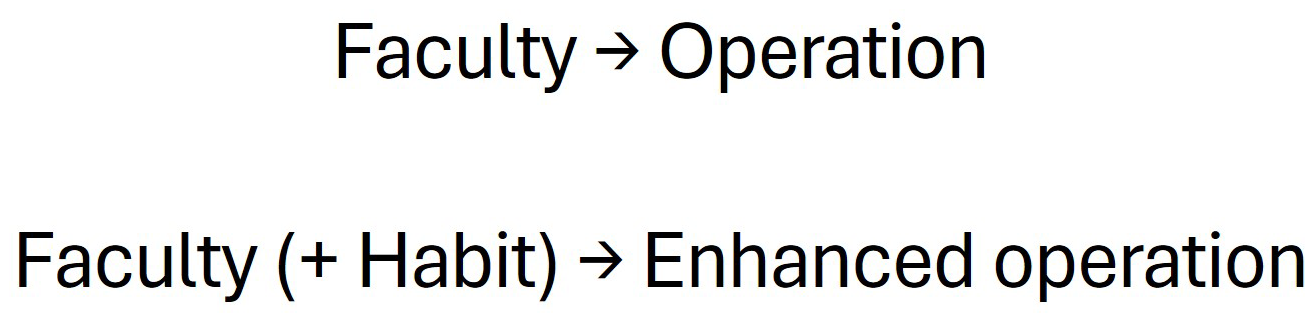
Relationship between operation, faculty and habit.

As was reviewed in the theoretical framework, the concept of ESR has been used to refer to different realities that, despite having some aspects in common, are not the same. In order to propose an orderly classification, we will use the Thomistic categories of *operation*, *faculty* and *habit* (see Figure 2). The operation, also called act, is any movement or change performed by an individual, whether external (e.g., breathing, walking, speaking) or internal (e.g., feeling, understanding). The faculty, also called *operative power*, is the capacity common to every human being to perform human operations (e.g., locomotion, senses, appetites and reason). This capacity can be actualized, as when an action is performed, or kept latent, as when we know that we can do something, although we are not performing it (Aquinas, 1920, I, c. 77, a. 6). Finally, the habit is a stable quality that the faculty can acquire, which enables the individual to perform its operation when he wants to (Aquinas, 1920, I-II, c. 51, a. 1), outstandingly, with promptitude and delight (Aquinas, 1999, a. 1) or, in some cases, at least “without sadness” (Id, a. 10, ad. 15). It is necessary to admit this notion because although all human beings can perform the same kind of operations (since we possess the same faculties), only some develop qualities to operate more perfectly. For instance, we are all capable of reasoning, but only some develop *science*, that is, the skill to reason with rigor and fluency in a field of knowledge.

Based on this scheme, it is necessary to distinguish three different realities that could be called ESR (see Table 2). First, the operation of ESR, i.e., the activity that an individual performs to regulate his emotions at a given moment. This activity should not be seen as an isolated act, but rather as a process involving multiple operations (Gross, 2015b). Second, the ESR faculty, i.e., the common capacity that allows human beings to regulate their emotions. Third, the habit of ESR, i.e., the acquired skill that enables an individual to regulate his emotions in an outstanding manner, with promptitude and delight.

**Table 2:** ESR categories derived from Thomistic categories.

Thomistic category	ESR categories	Definition	Example
Operation	ESR operation	Activity that an individual undertakes to regulate his emotions at a given moment.	The tennis match winner puts himself in his opponent’s shoes and moderates his joy so as not to offend him.
Faculty	ESR faculty	Common capacity that allows human beings to regulate their emotions.	At a certain point in development, children become capable of calming themselves.
Habit	ESR habit	Acquired skill that allows an individual to regulate his emotions in an outstanding manner, with promptitude and delight.	A fraudster speaks so calmly that he makes everyone believe he is telling the truth.

This distinction is relevant because the ESR categories do not necessarily occur together in the same individual. For instance, a person with emotion regulation skills might occasionally be driven by his emotions without regulating them. It could also happen that someone without ESR skills performs an ESR act if he prepares for it and makes an effort to do it. However, there are also individuals who, having the faculty to regulate their emotions, do not develop ESR skills and do not perform ESR acts.

Following this scheme, the appropriate definition of ESR would depend on the reality to be pointed out. However, when reviewing the definitions of ESR proposed in the literature, we realize that they do not seem to refer to the same reality. This could explain the difficulties in agreeing on a definition of the term. Table 3 shows some definitions of ESR and their possible relationship with our categorization. As can be seen, most of the definitions seem to understand ESR as an operation, only one as a faculty, and none as a skill.

**Table 3:** Definitions of ESR and their possible categorization.

ESR designation	Definition	ESR category
Emotional control	“Emotional control means both a turning toward what is truly lovable from a human point of view and a turning away from things that exert too strong a pull” (Arnold, 1960, p. 278).	Operation
Self-regulation of emotion	Self-regulation of emotion means “control not only over the overt behavior associated with an emotion (e.g., the expressive gestures and postures and instrumental action) but of the entire organized state that is subsumed under the emotion construct” and it also “dampens, eliminates, or alters the quality of emotional states” (Lazarus, 1973, pp. 172–173).	Operation
Emotion regulation	“Emotion regulation refers to the processes by which individuals influence which emotions they have, when they have them, and how they experience and express these emotions” (Gross, 1998, p. 275).	Operation
Self-regulation and Self-control	“We define self-regulation as processes by which the self intentionally alters its own responses, including thoughts, emotions, impulses, performance, and behaviors, based on standards” (Baumeister and Vohs, 2016, p. 68).	Operation
Self-regulation	“We can describe self-regulation as a largely unconscious form of volition that involves, and yet goes beyond, the integrative intelligence of motives. Volitional self-regulation draws not only on those networks of experiences that are relevant for one’s needs but on all autobiographical experiences that have contributed to the development of a coherent self-image” (Kuhl, 2018, p. 544).	Faculty

Arnold (1960) understands the process of emotional control from the Thomistic model, but delves only into the operation itself of what is involved in approaching or withdrawing from an object from the *appraisal* of the *cogitative*, and does not present aspects of ESR as a faculty or habit. Lazarus focuses on the operation of coping, but distinguishes between the place of appraisal, which in the Thomistic model comes from the *cogitative*, and the role of knowledge, which is related to the faculty of reason as it focuses on general judgment. Gross’s model emphasizes the processes of emotional generation and regulation, which can be understood as a concatenation of operations. On the other hand, the elements described in the *Modal Model of Emotion* (Gross, 2008) seem to be closely related to the Thomistic model, insofar as there is an object (situation) to which the senses are directed (attention), which leads to an initial and automatic evaluation of the object by the *cogitative* (appraisal), and which results in a movement of the passions toward that object (response). What would be missing in the *Extended Process Model of the Emotional Regulation* of Gross (2015b) is the role of reason and will in emotional regulation and the role of memory, the explanation of which is beyond the focus of this article.

The intention to include the will in the ESR is observed in both Baumeister’s and Kuhl’s models. The first understands the will from his SMSR as willpower, where any successful self-regulation implies a concatenation of operations involving effort and energy expenditure, organized by a central governor (Baumeister and Vohs, 2016). The second, on the other hand, understands the will from the PSI “as a set of central executive processes that regulate the person’s thoughts, feelings, and actions in a top-down manner” (Kuhl and Koole, 2004, p. 422), where the two volitional modes of self-control and self-regulation are distinguished, each with a different system of governance from the will, where the category of ESR faculty is expressed from the Thomistic model. Thus, it is seen that it is the will that orders regulation, either as an inner-democracy (Kuhl, 2000) or like a monetary authority (Baumeister and Vohs, 2016).

For his part, Thomas Aquinas considers that the will performs three types of acts related to what is for the end: *choice, consent* and *use* (Aquinas, 1920, c. 13, intro). All three can be applied to the act of ESR. First, the will *consents* to those things that reason deliberates as appropriate (Id., c. 15, a. 3), e.g., that in this situation it is convenient to self-regulate in certain ways. Next, the will *chooses* some of the alternatives previously consented to (Id., c. 15, a. 3, ad. 3), e.g., that it is convenient to regulate this anger by trying to understand the one who offends me. Finally, the will *uses*, that is, it moves the other powers to perform some act (Id., c. 16, a. 1), for example, moves reason so that it commands the act of the sensitive appetite.

Now, Thomas Aquinas considers that, strictly speaking, reason is the faculty that regulates the emotions (Aquinas, 1920, I-II, c. 17, a. 7). Indeed, he considers reason as the central governor who rules over the passions “not by a ‘despotic supremacy’, which is that of a master over his slave; but by a ‘politic and royal supremacy’, whereby the free are governed, who are not wholly subject to command” (*Ibidem*). This does not mean that the will is unrelated to emotional regulation, because “command is an act of the reason presupposing, however, an act of the will” (Aquinas, 1920, I-II, c. 17, a. 1). In other words, reason is the faculty that properly regulates the passions, but it is the will that consents and chooses to regulate the emotions, and then moves reason to perform this activity.

Interestingly, no definition refers directly to SRE as a habit. However, it would be premature to conclude that this is due to researchers’ disinterest in this reality. In fact, several ESR theories refer directly or indirectly to people’s acquired ability to regulate their emotions. SMSR, for example, argues that ESR is like a trainable muscle (Baumeister, 2016). It is possible that the lack of definitions of ESR habit is simply due to a lack of interest in working out precise definitions, or simply because it seems too obvious to make explicit.

### 3.2 How is the relationship between ESR and personality?

To answer this question thoroughly, we must relate the three proposed categories of ESR to personality. However, personality is also a concept that has been approached in many ways, as evidenced by the dozens of definitions that have been proposed (Engler, 2009; Funder, 2015; Larsen and Buss, 2017; Cervone and Pervin, 2022). Knowing that there are many valid models, in this manuscript we will approach personality from the FFM, which is highly acknowledged in the personality literature (Roberts and Yoon, 2022), and from the virtues model, which is rooted in the Aristotelian-Thomistic tradition. The first approach views personality as composed of five broad dimensional traits with strong biological roots, whose basic tendencies are manifested in characteristic adaptations (McCrae and Costa, 1996). Like most personality models, it considers that “individuals are born with the rudiments of personality dimensions and that those dimensions generally unfold without intentional effort over time” (Fowers et al., 2023, p. 4). The second considers that people acquire stable qualities in the interaction of biological, environmental and personal factors, among which intentional effort to achieve what the individual understands as happiness stands out. The relationship between the two models is not yet fully clarified (for a review, see Fowers et al., 2023), so they will be considered separately.

#### 3.2.1 The influence of personality on ESR as a habit

Let us start by reviewing the relationship between personality and ESR as a habit. Several authors have noted the correlation between personality traits and ESR using the FFM model. Specifically, they have found a significant association between high levels of Conscientiousness and ESR (Roberts et al., 2005b; Hoyle, 2006, 2010; McCrae and Löckenhoff, 2010; Barańczuk, 2019). This link is evident when considering the facets belonging to this trait: *competence/self-efficacy*, *orderliness*, *dutifulness*, *achievement striving*, *self-discipline*, and *deliberation/cautiousness*. On the other hand, an association has been found between low levels of Neuroticism and ESR (McCrae and Löckenhoff, 2010; Barańczuk, 2019). In turn, one of the aspects of this trait, *impulsiveness*, has been linked to low self-control (Hoyle, 2006, 2010).

However, when the relationship between FFM and Self-Regulation models is analyzed, it becomes clear that all traits play some role in the ESR. Extroversion is linked to *behavioral activation* and *sensation seeking*, which are significantly associated with lower *self-control*. Finally, although with less significance, *Openness* and *Agreeableness* are associated with ESR through *self-efficacy* and *behavioral inhibition*, respectively (McCrae and Löckenhoff, 2010).

Nevertheless, this link does not appear to be exactly linear. For instance, it has been found that too high levels of Conscientiousness can lead to a *maladaptive* ESR, highly rigid and overly persistent (Hoyle, 2006). In addition, it has been found that individuals with low Neuroticism and, at the same time, low Conscientiousness seem to have little interest in controlling their behavior (Costa and Piedmont, 2003). Other factors may mediate the link between personality and ERA. For instance, it has been proposed the importance of internal processes involving standards of behavior, the evaluation of one’s behavior about these standards, affective reactions to such evaluations, and mechanisms for correcting these gaps (Hoyle, 2006, 2010; Gross, 1998, 2015a, 2024; McCrae and Löckenhoff, 2010).

A similar complexity can be found in the work of Thomas Aquinas, who suggests that some people possess a natural predisposition for acts of virtue, which require emotional mastery. This is equivalent to affirming that certain traits, especially insofar as they have strong biological roots, have greater ESR facilities. However, the same author clarifies that this tendency is not sufficient to achieve the attainment of virtue:

There is another beginning of virtue which follows on individual nature, insofar as a man, by natural makeup or celestial influence, is inclined to the act of a given virtue. This inclination is a kind of beginning of virtue but is not perfected virtue, because for perfected virtue the governance of reason is needed, which is why the definition of virtue states that it is elective of the mean according to right reason. For if someone should follow such an inclination without the discernment of reason, he would frequently sin. Just as this beginning of virtue without the work of reason cannot have the perfect note of virtue, no more can any of the other beginnings of virtue mentioned (Aquinas, 1999, a. 8).

If we follow this analysis, reason would be responsible for mediating between innate personality tendencies and virtue. This faculty allows us to discern what is appropriate in each situation and also allows us to govern our emotions by putting in them their rule. To achieve this, reason moves the *cogitative*, the internal sense that judges stimuli as convenient or inconvenient for the vital interests of the subject (Aquinas, 1920, I, c. 78, a. 4). This evaluation, equivalent to the concept of appraisal (Arnold, 1960; Lazarus, 1991; Gross, 1998), triggers the emotions, which are nothing other than the movement of the sensitive appetite. In this way, the emotions can be considered open to reason (Aquinas, 1920, I, c. 81, a. 3) and subject to its rule (Aquinas, 1920, I-II, c. 17, a. 7), which means “regulated.” Consequently, although natural tendencies are principles of virtue, they cannot ensure the right operation of the faculty without reason.

Now, Thomas Aquinas explains that reason needs to be strengthened by the virtue of prudence, also known as *phronesis*, in order to investigate and judge what is appropriate in each circumstance and to execute it (Aquinas, 1920, II-II, c. 47, a. 8). Related to this, the current literature is addressing the high context sensitivity of ESR, because it tends to operate in the interaction between person, situation and strategy, as proposed by the interactionist framework of a *personalized science of emotion* (for a review, see Doré et al., 2016). From this perspective, in emotional regulation, both the context (for a review, see Aldao, 2013) and flexibility (for a review, see Kobylińska and Kusev, 2019) become particularly relevant in order to assess the effectiveness of ESR strategies. The close relationship between prudence and ESR was noted by Baumeister and Exline (1999), who stated “Prudence is obviously a matter of self-control” (p. 1174). To illustrate the similarities between this virtue and ESR it is useful to read Aquinas's description of the parts of prudence:

Of these eight, five belong to prudence as a cognitive virtue, namely, *memory*, *reasoning*, *understanding*, *docility* and *shrewdness*: while the three others belong thereto, as commanding and applying knowledge to action, namely, *foresight*, *circumspection* and *caution*. The reason of their difference is seen from the fact that three things may be observed in reference to knowledge. In the first place, knowledge itself, which, if it be of the past, is called *memory*, if of the present, whether contingent or necessary, is called *understanding* or *intelligence*. Secondly, the acquiring of knowledge, which is caused either by teaching, to which pertains *docility*, or by discovery, and to this belongs to *eustochia*, i.e., “a happy conjecture,” of which *shrewdness* is a part, which is a “quick conjecture of the middle term,” as stated in Poster. i, 9. Thirdly, the use of knowledge, in as much as we proceed from things known to knowledge or judgment of other things, and this belongs to *reasoning*. And the reason, in order to command a right, requires to have three conditions. First, to order that which is befitting the end, and this belongs to *foresight*; secondly, to attend to the circumstances of the matter in hand, and this belongs to *circumspection*; thirdly, to avoid obstacles, and this belongs to *caution* (Aquinas, 1920, II-II, c. 48, a. 1).

Returning to Aquinas’s thoughts on the innate factors favoring the formation of habits, we find a text in which he states that any natural predisposition to a certain virtue is, at the same time, an obstacle to the attainment of another:

It should be said that there can be a natural inclination with respect to the object of one virtue but not with respect to all because the natural disposition which inclines to one virtue inclines to the opposite of another virtue. For instance, one naturally disposed to courage, which is the pursuit of the arduous, is less disposed to patience, which consists in restraining the passions of the irascible. Thus we see that animals naturally inclined to the act of one virtue are inclined to a vice contrary to another virtue, as the Eon who is bold is also naturally cruel. Such natural inclinations to this or that virtue suffice for animals who are incapable of achieving the perfect good according to virtue but pursue some limited good. But men are made to achieve the perfect good according to virtue and must therefore have an inclination to all the acts of virtue, which, since it cannot be from nature, must come from reason, in which are found the seeds of all the virtues (Aquinas, 1999, a. 8, ad. 10).

This exposition introduces a nuance not very present in contemporary literature: emotions can be divided into two broad groups and require different self-regulatory skills. Indeed, Thomas Aquinas, in line with Aristotle and taken up by Arnold (1960), admits two types of emotions: those that drive toward goods insofar as they are goods, and those that bring goods closer insofar as they are challenging to attain (Aquinas, 1920, I, c. 80, a. 2). The former correspond to the activity of the concupiscible appetite —love, desire and pleasure, and their opposites, hatred, rejection and displeasure— and the latter to the irascible —hope, audacity, their opposites, despair, fear, and anger, which has no opposite—. The concupiscible needs to be perfected through the habit of temperance, which regulates desires and pleasures (Aquinas, 1920, II-II, c. 141, a. 3). On the other hand, the irascible requires the habit of fortitude, which allows firmness in the face of pain and danger (Aquinas, 1920, II-II, c. 123, a. 11).

If we admit the need for two different skills to govern emotions, we would have to consider two types of ESR: one focused on moderating pleasurable emotions and the other on maintaining high spirits in the face of danger. This distinction has already been slipped into the literature on self-regulation, as when it is stated that self-control can inhibit —restrain impulsive behavior— or activate —initiating and sustaining effortful activity— (McCrae and Löckenhoff, 2010; Kuhl, 2018). Following Thomas Aquinas’s proposition that the disposition for some virtues is an obstacle to attaining others, we could argue that personality traits associated with ESR, understood as behavioral inhibition, could be inversely related to ESR, understood as activation. For instance, we might consider that an individual with high Conscientiousness is prone to inhibit pleasurable emotions that divert him from his goals. However, this does not mean he is simultaneously inclined to pursue challenging goals, especially those involving risk, uncertainty and a certain degree of improvisation. According to the literature, the latter seems more associated with individuals with high *Extraversion*, who tend to possess little self-control.

Let us now consider the relationship between the virtues model and the ESR understood as a habit. For this ESR to emerge, it is necessary to develop prudence, temperance and fortitude. First of all, *prudence*, which enables the human being to discern what is appropriate in each circumstance and, at the same time, to command over the appetites (Aquinas, 1920, II-II, c. 47, a. 8). However, this virtue alone is not sufficient for proper emotional regulation, for if the appetites are not well disposed to be governed by reason, then the act will be imperfect, for it will lack the required emotionality and force. Thomas Aquinas illustrates this with a metaphor: in the making of a work, the artist must be well disposed, that is, he must be skilled in what he does, but the instrument he uses must also be well disposed (Aquinas, 1920, I-II, c.56, a. 4). Following this image, the well-disposed instrument corresponds to the sensitive appetite, the faculty that enables human beings to experience affective tendencies toward the good, or in other words, that allows them to feel emotions. This faculty is divided into concupiscible and irascible. As explained above, the first needs *temperance*, and the second needs *fortitude* in order to be able to follow reason with docility.

The incorporation of justice as a necessary virtue for the SRE is debatable. Indeed, we need it to prefer the common good to the individual good, from which stems much of the human motivation to self-regulation (Aquinas, 1920, I-II, c. 56, a. 6). Moreover, justice is attributed to the order of external operations (Aquinas, 1920, c. 61, a. 2). However, the role of the will in the ESR, according to the Thomistic scheme, is antecedent to the command of reason. It could be said that the will explains the motivation to perform acts of SRE, but it is not part of those acts. Thus, people with diverse motivations are capable of self-regulating their emotions, which includes virtuous motivations —such as doing good to others—, vicious motivations —such as stealing without being caught— or morally neutral motivations —such as sailing or climbing mountains—. This is not to exclude the role of the will in SRE, since, as we shall see, it is particularly relevant for understanding the self-regulation of the continent person. It is simply a matter of affirming that, strictly speaking, justice does not seem to be part of the SRE.

In addition to virtue, Thomas Aquinas, following Aristotle, postulates other moral habits. If *virtue* is the habit that orders the reason and the appetites toward the true good (Botkin, 1921), *vice* is the habit that orders them toward the apparent good or evil (Aquinas, 1920, I-II, c. 71, a. 1). In this case, reason is vigorised to perform evil, and the appetites are made docile to follow reason in its evil purpose, as in the case of the individual who is skilled in stealing. Arnold (1960) had already raised this difference when she commented that “such control of emotion implies a worth-while self-ideal to provide a focus for a man’s striving. A man may develop habits of right action (they used to be called virtues) or he may fall into habits of indulgence (formerly called vices)” (p. 278).

In addition to *virtue* and *vice*, the existence of an imperfect virtue, *continence*, is posited since it implies some perfection in reason but not in the appetites (Aquinas, 1920, II-II, c. 155, a. 1). In this case, the individual understands rationally what is proper and desires it voluntarily, but encounters an obstacle in his emotions, which inclines him to the contrary. Although he manages to follow the path indicated by reason, thanks to his will, his operations are neither harmonious nor satisfactory, as he experiences emotional tension. Finally, we have the lack of habit, *incontinence*, in which the individual also understands what is appropriate and desires it; however, the force of his passions habitually drags him to do what seems to him to be wrong. Thus, emotional satisfaction is followed by guilt, unlike the vicious individual who takes pleasure in his actions.

From these moral habits, one can proceed to the elaboration of a true characterology, depending on which habit is the most predominant in the individual’s life. Inspired by Aristotle, Fowers (2008) is perhaps the first to incorporate this characterology into the contemporary psychological literature. He also includes *bestiality*, which would be equivalent to the state of an individual habitually dominated by his emotions to such an extent that he cannot practically reflect or take conscience of his actions. The relationship of this characterology to the disposition of the psychic faculties —and to the ESR as a habit— can be seen in Table 4.

**Table 4:** Aristotelian character types, their relationship to psychic faculties and to ESR as a habit.

Character type	Reason	Sensory appetite	Will	ESR habit
Virtue	Right	Docile	Firm (effortless)	Perfect
Continence	Right	Indocile	Firm (effortful)	Imperfect
Incontinence	Right	Indocile	Weak	Faulty
Vice	Distorted	Docile	Firm	Maladaptive
Bestiality	Overruled by emotions	It is which governs	Weak	None

According to this scheme, only the virtuous would possess the perfect habit of ESR: his reason is rightly ordered, the sensory appetite is docile to reason, and his will is firmly determined toward the good. This enables him to regulate his emotions so that he acts *by reason and with emotion*. He is aware when he experiences disordered emotions and can redirect them without much difficulty, which fits well with the concept of *effortless willpower* (Quirin et al., 2021). This is somewhat similar to the self-regulatory system proposed by Kuhl et al. (2021), in that it operates at an implicit, experiential level, which allows for personality identification and integration (Ryan and Deci, 2019). The vicious person seems to possess similar self-regulation skills; however, his reason has lost its sense of what is good, and so he misreads reality and fails to grasp what is truly convenient. For this reason, his self-regulation will never be fully adaptive and may disintegrate personality: he acts *by distorted reason and with emotion*. The continent, on the other hand, has some degree of self-regulation; however, he depends on a will that operates in opposition to emotions. Therefore, its possibilities of inhibiting them are limited, which fits well with *effortful willpower* (Quirin et al., 2021). His ESR closely resembles the model of self-control as moral muscle (Baumeister and Exline, 1999) and the self-control system of PSI (Kuhl, 2018), leading him to act *by reason and against emotion*. The incontinent is weak-willed; therefore, when emotions arise with vehemence, he cannot resist. He fails to regulate his affections, which makes his behavior defective: he acts *by emotion and against reason*. Finally, the bestial character is so governed by his emotions that his reason has been overruled. Without the rule of reason, no emotional regulation is possible: he acts *by emotion*.

#### 3.2.2 The influence of personality on the ESR as an operation

The influence of personality on the operation of ESR is similar to its influence on the habit of ESR since habit is nothing but a stable disposition to operate in a certain way. Therefore, roughly speaking, the same conclusions apply to the ESR operation as to the habit. However, it is necessary to introduce a nuance. The personality traits proposed by the FFM model and the habits of the virtues model can be conceptualized as stable operative dispositions, i.e., habitual inclinations toward a certain activity. However, the relationship of dispositions is more robust concerning other dispositions than singular actions. An individual predisposed to acts of temperance is equally predisposed to acquiring the habit of temperance. However, for an individual poorly predisposed to these acts, it is easier to perform some isolated act of temperance than to acquire the virtue of temperance, for it requires many acts to form. Indeed, although less frequent, there is nothing to prevent individuals with low Conscientiousness from sometimes acting in a planned manner or, conversely, someone with low Neuroticism from sometimes being driven by their emotions. The reason is that, for the Thomistic system, temperament and personality are predispositions that condition action but do not determine it. Indeed, for Thomas Aquinas, the human being acted freely even under the most violent pressures of the environment and interfered with by one’s emotions: as long as there is some degree of use of reason, there is always a choice to be made (Aquinas, 1920, I-II, c. 6, a. 4).

This is even clearer for those who possess some virtue. Indeed, virtue does not force one to act virtuously since virtue is a habit, and as such, is “something we use when we will” (Aquinas, 1920, I-II, c. 78, a. 2). For this reason, it is inconceivable that the virtuous person lacks the habit of ESR. However, it is perfectly admissible that he performs some unregulated act in isolation.

#### 3.2.3 The influence of personality on ESR as a faculty

It does not appear that there is any personality influence on the occurrence or existence of the ESR faculty. In fact, all human beings possess the faculties involved in ESR, regardless of their personality traits, as Kuhl’s (2018) PSI model proposed. Perhaps it could be studied whether the ESR faculty appears or is consolidated in some individuals earlier than others; in that case, it could also be examined whether this is exclusively due to biological factors or whether there could be some influence of temperament or personality. However, once adulthood is reached, it is considered that everyone, regardless of their personality traits or moral habits, possesses the capacity to regulate their emotions. Otherwise, some individuals would be exempted from legal and moral obligations since no one can be required to do what he is incapable of.

The philosophical foundation is that every operative disposition is a quality of some faculty. Faculties are ontologically prior to dispositions, and therefore, no disposition can create or bring into being a faculty. On the contrary, the faculties support the existence of dispositions and are thus some kind of cause of them.

#### 3.2.4 The influence of the ESR as a faculty on the personality

ESR, understood as a common faculty or capacity, appears gradually in the individual from infancy, possibly as a function of brain development (Posner and Rothbart, 2000; Magen and Gross, 2010; McDermott and Fox, 2010). However, importance is also attributed to the environment, e.g., caregivers (Crocker and Park, 2004; Morf and Horvath, 2010). The emergence of this ESR has been postulated as a key factor for personality development (Rothbart, 1981; Posner and Rothbart, 2000; Blair et al., 2010; Denissen et al., 2013). It can be observed that children are more impulsive than adults and that Neuroticism scores tend to decrease from adolescence into adulthood (Terracciano et al., 2005). Although this decrease in Neuroticism trait may be explained by the acquisition of ESR skills, it is also possible that brain maturation plays an important role. Indeed, the prefrontal cortex, which is closely related to emotional self-regulation processes, is continuously developed from birth to early adulthood (Magen and Gross, 2010). It could also be proposed that the child’s early attachment to parents or caregivers plays a role, as ESR is taught by the regulatory activity of caregivers (Fonagy and Target, 2002). In addition, mentalization, which is also acquired thanks to caregivers, is seen as a critical step in the emergence of self-regulatory capacity (Schwarzer et al., 2021), which can be related to mentalized affectivity (Jurist et al., 2023).

According to the Thomistic scheme, virtues begin to develop at a very early age. Even if there is little use of reason or ESR, stable operative dispositions can be formed thanks to parents, who provisionally assume the role of reason (Palet, 2022). The development of the capacity for ESR, possibly simultaneously with the development of the capacity to reason, allows the individual to forge his habits by himself. In this way, the person begins to be the architect of his personality through his decisions.

#### 3.2.5 The influence of the ESR as an operation over personality

The ESR operation does not seem to influence personality. From the FFM model, at least as McCrae and Löckenhoff (2010) put it, traits are strongly biologically based, develop involuntarily (Fowers et al., 2023) and are hardly modifiable (Roberts et al., 2005a). Therefore, no act of ESR would have any impact on personality.

The Thomistic system considers that a single operation is not sufficient for the acquisition of any habit (Aquinas, 1920, I-II, c. 51, a. 3). Therefore, unless there are many of them, the act of ESR does not influence the personality either. Now, in one who has already developed some habit, each act of ESR aligned with his character would reaffirm the previous inclination. Thus, the ESR acts have some influence on personality.

#### 3.2.6 The influence of ESR as a habit on the personality

ESR as a habit is related to personality traits in two ways, depending on whether it is conceived as a skill included within personality traits or as a separate skill that interacts with them. Let us consider the first alternative. Several authors favor this option. McCrae and Löckenhoff (2010) have postulated that ESR would be implicated in the facet scales *Impulsiveness, Excitement seeking, Self-discipline,* and *Deliberation*. Fein and Klein (2011) postulated that ESR would be related to *assertiveness, activity, achievement striving, deliberation, dutifulness, self-discipline,* and *ideas*. However, these alternatives seem contrary to the Aristotelian-Thomistic position. If ESR were part of these personality traits, then it would share some properties common to all of them, such as being substantially influenced by genetics and essentially unrelated to the environment. In this case, character education would have minimal impact on personality traits, and thus also on ESR. This, as McCrae and Löckenhoff (2010) note, “is a startling conclusion, flying in the face of centuries of traditional wisdom and most accounts of personality functioning” (p. 161). By contrast, Aristotle and Thomas Aquinas consider education fundamental to acquiring virtues, and emotional mastery is undoubtedly a fundamental part of this acquisition.

Now, let us consider ESR habits as a separate skill from personality traits. This habit can be understood in two ways: as a perfect skill to modify affective states or as a skill to maintain appropriate behavior despite emotions (Fowers, 2008). In the first case, one could admit the influence of this habit on the rest of the personality since the habitual regulation of emotions would change its stable disposition. At least this is how Thomas Aquinas understands it, for whom the repetition of acts, for instance, of temperance, is capable of developing the virtue of temperance (Aquinas, 1920, I-II, c. 51, a. 2; c. 63, a. 2). In contrast, ESR understood as behavioral control would be an inferior form of ESR, just as continence is inferior to virtue, as discussed above. Although commendable, this type of ESR is more focused on the regulation of external behavior than on actual emotional change and, for that reason, focuses on emotional inhibition or suppression. As Baumeister and Vohs (2016) have noted, this control requires effort, and as the effort is limited, it eventually exhausts itself. If this type of ESR cannot modify emotions, then it is even less capable of modifying its habitual disposition. It is a matter of debate whether ESR, understood as self-control or continence, is a first step toward true emotion regulation, or whether it operates along a separate path and is therefore incapable of engendering long-term emotional change.

From the Thomistic point of view, the ESR understood as a habit, in the most profound sense, does not influence habits but is part of them. As explained above, ESR emerges when there is prudence, temperance and fortitude. It is unnecessary to conceptualize any new virtue to explain the individual’s habitual disposition to fully control his emotions. Some authors have proposed that ESR would be a meta-virtue or meta-habit, i.e., an independent disposition, which would not be part of the personality. This has already been proposed directly by some authors (e.g., Strauman and Wilson, 2010; Fowers et al., 2023), and indirectly by others, for whom ESR is part of prudence, understood as a meta-virtue (e.g., Kristjánsson et al., 2021). For this to be true, this meta-virtue would have to consist of the invigoration of some power, but as we have argued, the invigoration of only one power is not enough for the fullness of self-regulation. Formulating that prudence, temperance and fortitude have a regulative dimension seems more appropriate. Indeed, prudence encompasses several acts, but not all are related to the ESR. Temperance and fortitude are eminently emotion-regulating virtues but focus on different types of emotions. In short, the ESR would not be strictly speaking a habit but a set of habits that include the part of prudence dedicated to the rule of appetites and the common capacity of temperance and fortitude to regulate emotions.

All this applies to understanding the habit of ESR as a perfect virtue. However, we can also understand it as an imperfect virtue, as in the case of continence. In this case, the ESR is partial because it only includes the conducting dimension of prudence but lacks the docility of the appetites. As its name says, the continent contains the emotional and behavioral expression of its emotions; he does not order them. Insofar as it is not the emotion itself that is regulated but rather the behavior, this type of regulation could be called behavioral self-regulation.

## 4 Discussion

The interdisciplinary dialogue, specifically with Thomistic anthropology, has not only allowed us to answer the major questions posed at the beginning but also to clarify some of the difficulties pointed out in the introduction. Firstly, with regard to terminology, it seems important to propose moving toward a common use of concepts, or at least toward making explicit the realities that we are signaling with them (Quirin et al., 2021). It is confusing when different terms point to the same thing or when a single term indicates different realities. Following important definitions of ESR, such as the one proposed by Gross (2015b), we have detected at least three realities that could be called ESR, which are sufficiently different to justify the search for greater terminological precision. The distinction between operation, faculty and habit has proven to be helpful for this purpose. It is possible that this distinction was evident in the minds of some scholars; if so, this article represents an advance by explicitly systematizing it.

Another contribution of the Thomistic approach to the understanding of ESR is its moral dimension, which was introduced by Arnold and Gasson (1954) 70 years ago, but tends to be little incorporated in the specialized literature. To exemplify this point, it is noticeable that there is a considerable difference between the skill of a pupil to simulate a severe cold in order to evade a test and that of a classmate who refrains from cheating when he remembers the kind of person he wants to become. Both students, indeed, share a particular skill to regulate their emotions. At the same time, however, we understand that there is a wide gap between them: a good educator will be sad for the first and proud of the second. We could go deeper and ask whether the first’s cowardice might be the expression of a poor skill to self-regulate negative emotions such as fear of getting a bad grade or embarrassment at possible parental reprimand. In any case, especially if we focus on adolescents, the relationship between emotional regulation and concepts such as psychological maturity or human flourishing could be raised, which is far beyond the scope of this manuscript but has been reviewed in other studies (e.g., Barber et al., 2010; Richard-Sephton et al., 2023).

If the concept of ESR is complex, its connection to personality is even more. Given the scope of the subject, in this article we have restricted ourselves to the FFM, which considers personality as a set of unintentional qualities with a strong biological basis, and to the virtues model outlined by Aristotle and reordered by Fowers (2008), which considers the qualities acquired in the interaction between biology, environment and rationality.

Within the first model, it is important to highlight the relationship that has been found between some personality traits and ESR. However, whether these studies considered ESR as perfect emotional self-regulation or self-control in impulse restraint is unclear. Some clues suggest the second alternative, as when it is argued that high Consciousness can lead to an excess of control that prevents appropriate spontaneity of behavior (Hoyle, 2006, 2010). From the Thomistic point of view, such a trait could not be considered a perfect virtue, since it implies the proper adjustment of emotionality and behavior with respect to reality. Inhibition as a habitual mechanism seems closer to the notion of continence, which is admirable in that it curbs maladaptive tendencies but is imperfect in that it fails to achieve affective order. Reviewing the literature to establish whether virtue or continence has been studied is necessary.

In addition, we have seen that a series of cognitive processes modulate the relationship between personality traits and ESR. We have already hinted at the possible relationship that could be drawn between these processes and the qualities that make up prudence. Our proposal is that reason plays an important role in ESR. If so, the connection between the FFM’s personality traits and the habit of ESR may not be so intense or even direct since the rational factor would be preponderant. This consideration overcomes hasty conclusions that might link ESR only to certain personality types, excluding the importance of education. If reason is essential, then all individuals, regardless of their personality traits, are suited to regulate their emotions successfully. This does not detract from the fact that some personality traits facilitate ESR. However, as we pointed out, the ease of regulating some emotions may imply a difficulty in regulating others. In sum, innate dispositions are relevant to acquiring ESR as a habit, but the critical factor would be reason.

It is true that some authors have already mentioned that ESR includes not only processes of affective moderation but also affective drive and maintenance (Gross, 2024). However, the literature seems much more focused on ESR, understood as the former. The Thomistic system can significantly help to adequately conceptualize this difference, providing valuable conceptual tools to systematize and advance the study of the second type of ESR. The concupiscible appetite, which tends toward what is convenient in the sensible order, and shuns what is harmful, is the one that must be perfected by temperance so that emotions do not distract the person from his purpose. On the other hand, the irascible, which rejects everything that opposes it in the pursuit of what is convenient and detrimental to it, is perfected by fortitude, enabling it to persevere in its purpose despite difficulties. When we consider things this way, it opens up the possibility that some personality traits associated with a lack of ESR may be associated with emotional regulation in situations requiring boldness, bravery, use of aggression, risk, activity, speed, strength, etc.

The distinction between *virtue* ESR and *continence* ESR is also relevant when considering how ESR influences personality. Only the first one would be capable of modifying the personality. This has some therapeutic implications. In fact, if psychotherapy involves some degree of modification of affective dispositions, then only the ESR that modulates emotions would be truly capable of producing change. In contrast, techniques related to impulse inhibition might have some practical utility but would not directly help to improve any disposition. As such, they could even have a negative effect in the long run, as psychotherapy based on impulse inhibition techniques would end up frustrating patients’ hopes for real change in their way of feeling.

Perhaps it is no coincidence that the Person-Oriented Regulation Models described in the theoretical framework dialogue with similar anthropological currents, as does Personality Systems and Interactions (PSI) (Kuhl, 2000) with Aristotle, and Strength Model of Self-Regulation (SMSR) (Baumeister and Exline, 1999) with Thomas Aquinas. In particular, it is remarkable that to describe their models, they use analogies of political systems, such as “inner dictatorship” for the PSI self-control system, “inner democracy” for the PSI self-regulation system, and “central governor” for SMSR. This characteristic could echo Aristotelian-Thomistic philosophy, which proposes a “political government” of reason over emotions to guide the person toward developing his personality (Arnold, 1960).

The characterological scheme of Aristotle, replicated by Fowers (2008), seems particularly interesting in understanding the relationship between personality and ESR, understood in the broad sense of a set of stable operative dispositions. Each of the five traits relates to the ESR in a different way. The nuances of this scheme reveal the complexity of the human soul grasped by the Greek philosopher and taken up by Aquinas in the 13th century. Undoubtedly, this scheme has much to contribute to understanding the different types of ESR and can be a tremendous contribution to future research.

Finally, it seems necessary to highlight the idea put forward at the end of the interdisciplinary dialogue. ESR, understood as a habit, is not fully identified with Aristotelian virtues. As we explained, it requires stable dispositions of reason, will and sensitive appetites. By positing that the ESR requires the coordinated action of several powers, it becomes more evident that it is a coordinated set of habits. In other words, it is a system of rational and appetitive dispositions that harmonize emotions according to the rule of reason. On the other hand, it cannot be considered part of the personality, as understood in the light of the FFM model. We have already explained our arguments: ESR cannot be a biologically based trait that occurs unintentionally. However, if we consider personality as the total set of operative dispositions, including intentional and unintentional ones, it could be considered part of it. In this case, the ESR would be part of the personality, not a trait nor a single habit, but a set of acquired habits, less or more facilitated by the temperamental traits of the personality.

## 5 Conclusion

This article has reviewed the concept of ESR and its connection to personality through the interdisciplinary dialogue between psychology and Thomistic anthropology. We hope our conclusions will help achieve a fine conceptualization of the ESR and its categories, which could also improve the associated empirical studies. Indeed, a good conceptualization favors a better operationalization. Although our aim is theoretical, in the sense that we propose to rethink the existing literature from an interdisciplinary paradigm, it is clear that our postulates will need to be proved in order to increase their legitimacy.

We have proposed that the link between ESR and personality can be understood in many ways. However, regardless of the different meanings of these concepts, it seems clear that it is possible to establish highly relevant, two-way relationships. This brings us back to the seminal contribution of Arnold and Gasson (1954) and Arnold (1960), a pioneer in the generative model of emotion, who noted early on that emotional control is always executed in pursuit of some personality-related goal. As we have already discussed, this has profound implications for the therapeutic field. With ESR being so central to personality development and the achievement of therapeutic goals, it seems appropriate to join the voices proposing its inclusion in psychotherapy, especially in diagnosis and clinical intervention (e.g., Dadomo et al., 2016; Fassbinder et al., 2016; Grecucci et al., 2016, 2017; for a review, see Gratz et al., 2015).

The Thomistic model is particularly suited to dialogue with the psychology of the ESR and personality. The depth of the Italian thinker and the magnitude of his work have not lost their relevance; on the contrary, we are witnessing a greening of his thought, which coincides with the 800th anniversary of his birth. This can be corroborated in the field of psychology (e.g., Echavarría, 2005; Cornelius, 2006; Dryden, 2016; De Haan, 2019; Navarini and de Monte, 2019; Cartagena, 2021; Asociación de Psicología Integral de la Persona, 2022; Cubillos, 2022; Droste, 2022; Rojas Saffie, 2022; Schell, 2022; Suazo, 2022; Verdier, 2022; Marple et al., 2024). We hope that this manuscript will contribute to the field of ESR and serve as an inspiration for the continuation of this fruitful interdisciplinary dialogue.

## Data availability statement

The original contributions presented in the study are included in the article/supplementary material, further inquiries can be directed to the corresponding author.

## Author contributions

JR-S: Conceptualization, Investigation, Methodology, Project administration, Supervision, Writing – original draft, Writing – review & editing. NG-M: Conceptualization, Investigation, Writing - original draft, Writing - review & editing, Visualization.

## Footnotes

^1^Admittedly, this concept is not completely equivalent to emotion (Dryden, 2016), but they are similar enough to be considered interchangeable in this article.
